# A review of the effect of omega-3 polyunsaturated fatty acids on blood triacylglycerol levels in normolipidemic and borderline hyperlipidemic individuals

**DOI:** 10.1186/s12944-015-0049-7

**Published:** 2015-06-06

**Authors:** Michael A. Leslie, Daniel J. A. Cohen, Danyelle M. Liddle, Lindsay E. Robinson, David W. L. Ma

**Affiliations:** Department of Human Health and Nutritional Sciences, College of Biological Science, University of Guelph, Animal Science/Nutrition Building, Room 342, 491 Gordon Street, Guelph, ON N1G 2 W1 Canada

**Keywords:** n-3 PUFA, Cardiovascular disease, Normolipidemic, Cholesterol, Triacylglycerol

## Abstract

Circulating levels of triacylglycerol (TG) is a recognized risk factor for developing cardiovascular disease, a leading cause of death worldwide. The Institute of Medicine and the American Heart Association both recommend the consumption of n-3 polyunsaturated fatty acids (PUFA), specifically eicosapentaenoic acid (EPA) and docosahexaenoic acid (DHA), to reduce serum TG in hyperlipidemic individuals. Additionally, a number of systematic reviews have shown that individuals with any degree of dyslipidemia, elevated serum TG and/or cholesterol, may benefit from a 20-30 % reduction in serum TG after consuming n-3 PUFA derived from marine sources. Given that individuals with serum lipid levels ranging from healthy to borderline dyslipidemic constitute a large portion of the population, the focus of this review was to assess the potential for n-3 PUFA consumption to reduce serum TG in such individuals. A total of 1341 studies were retrieved and 38 clinical intervention studies, assessing 2270 individuals, were identified for inclusion in the current review. In summary, a 9-26 % reduction in circulating TG was demonstrated in studies where ≥ 4 g/day of n-3 PUFA were consumed from either marine or EPA/DHA-enriched food sources, while a 4-51 % reduction was found in studies where 1–5 g/day of EPA and/or DHA was consumed through supplements. Overall, this review summarizes the current evidence with regards to the beneficial effect of n-3 PUFA on circulating TG levels in normolipidemic to borderline hyperlipidemic, otherwise healthy, individuals. Thus demonstrating that n-3 PUFA may play an important role in the maintenance of cardiovascular health and disease prevention.

## Introduction

Cardiovascular disease (CVD) is one of the leading causes of mortality in North America, accounting for 1/3 and 1/6 of all deaths in Canada and the United States, respectively [1, 2]. Elevated levels of circulating triacylglycerol (TG) have been identified as an independent risk factor for developing CVD; evidence from Hokanson *et al.* indicates that an 88 mg/dL increase in fasting TG levels elevates the risk of developing CVD by 14 % and 37 %, in males and females, respectively [3–8]. Additionally, a large proportion of Canadians and Americans (26 % and 14 %, respectively) have been reported to be either hypertriglyceridemic or hyperlipidemic [2, 9, 10]. Omega 3 (n-3) polyunsaturated fatty acids (PUFA) have well-established TG lowering effects in hyperlipidemic individuals which may extend to normolipidemic populations [11–19].

The 2002 Institute of Medicine (IOM) report on Dietary Reference Intakes for various macronutrients, including fat, states that, “Supplementation with fish oil, which is high in EPA and DHA, reduces triacylglycerol concentrations; low density lipoprotein cholesterol and high density lipoprotein cholesterol concentrations are either increased or unchanged” [11]. Food and supplement based studies assessing the lipid-lowering effects of n-3 PUFA have utilized a variety of oils comprised of either flaxseed-derived alpha-linolenic acid (18:3 n-3, ALA), algal-derived pure eicosapentaenoic acid (20:5 n-3, EPA) or docosahexaenoic acid (22:6 n-3, DHA), or some combination of these n-3 PUFA through the consumption of fish or fish oils. It is therefore important to determine which forms of n-3 PUFA, or combination of forms, are bioactive in affecting serum TG levels and to detect differences in efficacy among various forms.

Systematic reviews by Wei and Jacobson, Bernstein *et al.*, and Eslick *et al.* highlight the lipid-lowering capacity of different sources and forms of n-3 PUFA in populations that range from normolipidemic to hyperlipidemic [20–22]. Wei and Jacobson compared the efficacy of DHA to EPA and found that individuals who consumed either DHA or EPA experienced reductions in serum TG of 18.5 % and 32 %, respectively. Individuals also exhibited significantly elevated serum low density lipoprotein cholesterol (LDL-c) levels by 5 % after DHA consumption, while those consuming EPA had a non-significant reduction in serum LDL-c of 1 % [20]. Additionally, Bernstein *et al.* concluded that DHA from algal oil plays a role in reducing serum TG while elevating serum LDL-c [21]. The results of a systematic review by Eslick *et al.* showed that 3.25 g/day of fish oil (1.9 g of EPA and 1.35 g of DHA) reduced serum TG by 14 %, yet cholesterol levels were not altered beyond a clinically insignificant increase in LDL-c [22]. Similar results were obtained in systematic reviews by Balk *et al.* and Mori *et al.*, both of which assessed studies in which individuals consumed either fish, algal EPA or algal DHA oils [23, 24]. While results from the previously identified systematic reviews indicate that DHA contributes to slight increases in LDL-c and EPA contributes to minor reductions in LDL-c, both n-3 PUFA were established to be efficacious in significantly lowering serum TG levels.

The previously discussed meta-analyses and systematic reviews concur with the observation that EPA and DHA, derived from fish or algal oils, can reduce serum lipids, most notably TG. However, these analyses primarily focused on hyperlipidemic individuals, a population likely to achieve the most drastic reduction in TG levels upon n-3 PUFA consumption, and may conceal a lack of response in normolipidemic subjects, whom with they were pooled [18]. The purpose of the current review is to provide new knowledge with regards to our understanding of the effect of dietary and supplemental n-3 PUFA intake on blood lipid profiles in healthy individuals, using the 2002 IOM report as a reference point. A focus will be placed on the TG-lowering ability of n-3 PUFA in normal to borderline hyperlipidemic populations as defined by AHA guidelines.

## Methods

### Search strategy

**Fig. 1:**
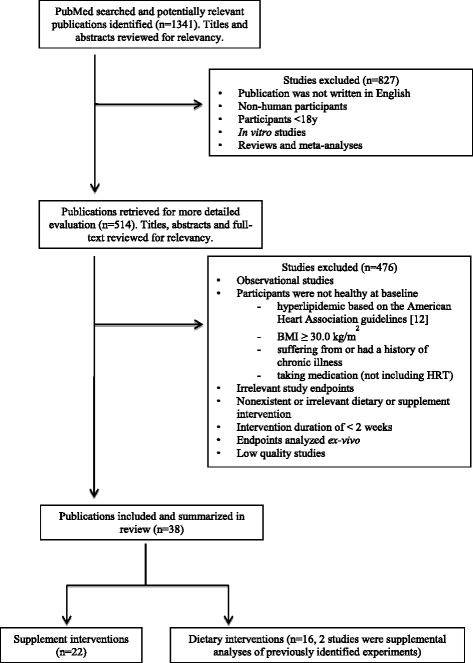
Flow chart of the study selection and exclusion methodology

Using the PubMed search engine, clinical trials and observational studies relating to the effects of n-3 PUFA on serum biomarkers of CVD were collected. Search terms (described below) were used to gather studies published from January 1, 2000 – October 1, 2013 as they were not captured within the reference point of the 2002 IOM report. Search terms relating to cholesterol, C-reactive protein (CRP) and chronic illnesses were used to capture the entirety of literature analyzing blood lipids as such studies would likely have included TG measurements (Fig. 1 summarizes the search strategy).

***Search terms:***

1. n-3 **OR** omega 3 **OR** EPA **OR** eicosapentaenoic acid **OR** DHA **OR** docosahexaenoic acid **OR** ALA **OR** alpha-linolenic acid

***AND***

(2) dyslipidemia **OR** total cholesterol **OR** cholesterol **OR** LDL cholesterol **OR** HDL cholesterol **OR** triacylglycerol **OR** CRP

***AND***

(3) heart disease **OR** CVD **OR** cardiovascular disease **OR** stroke **OR** diabetes **OR** obesity

This search returned 1341 results on PubMed. An initial screen of the title and abstract for relevancy was conducted based on the following inclusion and exclusion criteria:

***Inclusion criteria***

- Published between 1/1/2000 and 1/10/2013
- Human participants
- Article written in English
- Adult participants aged >18 years

***Exclusion criteria***

- *In vitro* studies
- Participants were suffering from or had a history of chronic illness (i.e. CVD, type 2 diabetes, cancer, etc.)
- Participants were medicated – not including hormone replacement therapy (HRT)
- Reviews and meta-analyses

Based on the above, 514 published studies (collected by two independent researchers) were reviewed in detail and 207 clinical trials were identified for further consideration. The final list of clinical trials was selected based on the following inclusion and exclusion criteria:

***Inclusion criteria:***

- Healthy participants, defined as:
- Healthy or moderately hyperlipidemic participants according to the American Heart Association (AHA) guidelines [plasma lipid levels for borderline hyperlipidemic individuals are: 200 ≤ total-cholesterol (total-c) ≤ 240 mg/dL, 130 ≤ LDL-c ≤ 160 mg/dL, and 150 ≤ TG ≤ 200 mg/dL] [14, 25]
- Healthy or overweight participants: Mean BMI within intervention group(s) and control/placebo group (if applicable) at baseline ≤29.9 kg/m^2^
- Study end-points included blood lipid parameters (TG, total-c, high-density lipoprotein cholesterol (HDL-c), LDL-c)*
- Study must involve a dietary or supplement intervention with n-3 PUFA
- Study duration was at least 2 weeks

***Exclusion criteria***

- Participants that fulfilled the inclusion criteria were analyzed with participants that were explicitly stated to have not met the inclusion criteria
- Endpoints were analyzed *ex vivo*
- Low quality studies**

*Studies that did not report a baseline profile for total-c, LDL-c and/or TG were included in analysis if they fulfilled all remaining inclusion and exclusion criteria described above.

**Study quality was assessed by the Appraisal guide for intervention/experimental studies provided by Health Canada [26], if studies scored below a threshold of seven they were excluded from the current review (a single study scored below this threshold).

Applying these parameters yielded 38 clinical trials that were further stratified into dietary interventions (16 studies) and supplementation trials (22 studies).

## Results

### Dietary interventions

Using the indicated selection criteria, 16 dietary intervention studies (633 subjects in total) were deemed appropriate for the assessment of the impact of n-3 PUFA consumption on circulating TG and cholesterol levels within normolipidemic and borderline hyperlipidemic (otherwise healthy) adults (Table 1). It is noteworthy that two of the 16 studies [27, 28] were subsequent analyses of formerly identified interventions [29, 30]. Therefore, they are presented as single experiments in this review. As a result, a final list of 14 unique dietary intervention studies were assessed (Table 1). Among the 14 dietary intervention studies, four studies utilized increased fish consumption [31–34]**;** five studies examined the benefits of increasing EPA/DHA intake by enriching foods to contain a higher content of n-3 PUFA [27, 29, 35–38]; three studies measured the effects of increasing ALA intake [28, 30, 39, 40]; and 2 studies altered the n-6:n-3 PUFA ratio while maintaining a constant amount of n-3 PUFA consumption [41, 42]. Alterations from baseline plasma TG and cholesterol values were assessed in all 14 intervention studies in the current review. Overall, of the 24 experimental arms within the 14 studies, 15 showed reduced serum TG levels (five were statistically significant reductions; Table 2) and eight of the 14 studies showed reduced serum total-c, LDL-c or both (five were statistically significant reductions; Table 1). The three dietary interventions that provided ≥ 4 g/day of EPA and/or DHA noted significant reductions in TG of 9-26 % [27, 29, 31, 33], while six out of seven experimental arms providing 2.3-3.4 g/day of marine based n-3 PUFA produced non-significant reductions in TG of 3-14 % [32, 34, 42], and the effect of n-3 PUFA provided from flaxseed, flaxseed oil or ≤ 2 g/day of EPA and/or DHA remains ambiguous [28, 30, 35–40].

**Table 1:** Studies assessing the lipid lowering effects of n-3 PUFA by dietary intervention

Study	Subject Characteristics	n-3 PUFA Source (~dose/day)	Study Design	Duration	Lipid Outcomes	Other Findings
	*Average baseline TG, Total-C, LDL-C (mg/dL)*					
Lara (2007) [31]	16 males, 32 females	125 g of salmon (5.4 g of n-3 PUFA)	Intervention (no placebo)	4 week intervention; 4 week washout without fish	TG reduced 15 % (sig)	Blood pressure reduced 4 % (sig)
	Scottish				LDL-c reduced 7 %	
	20–55 yrs old					
						Adiponectin reduced
	*(TG – 83, Total-C - 167, LDL-C - 92)*				HDL-c elevated 5 % (sig)	
Hallund (2010) [32]	68 males	150 g of trout fed marine diet (3.4 g n-3 PUFA)	Randomized, parallel arm trial	8 weeks	TG reduced 14 % and 6 % in participants consuming trout fed marine-based diet and trout fed a vegetable-based diet, respectively	Trout fed marine-based diet resulted in a reduction of blood pressure and CRP, compared to trout on vegetable diet
	Danish					
	40–70 yrs old					
	*(TG – 102, Total-C - 189, LDL-C - 117)*	Vs.				
		150 g of trout fed vegetable diet (0.8 g n-3 PUFA)				
Ambring (2004) [33]	12 males, 10 females	Mediterranean diet (4.1 g n-3 PUFA)	Randomized, cross-over trial	4 week on one diet, 4 week washout, 4 week on opposite diet	TG reduced 9 % in the group receiving Mediterranean diet	Consumed fewer calories on Mediterranean vs. Swedish diet (1869 vs. 2090, respectively)
	Swedish					
		Vs.				
	30–51 yrs old	Swedish diet (2.3 g n-3 PUFA)			Switching from a Swedish diet to a Mediterranean diet reduced serum TG, Total-c and LDL-c by 17 %, 17 % and 23 %, respectively (sig)	
	*(TG – 97, Total-C - 217, LDL-C - 139)*					
		*Source of n-3 PUFA in both diets was oily fish*				
Navas-Carretero (2009) [34]	25 iron deficient females	Oily fish diet (2.8 g n-3 PUFA)	Randomized, cross-over trial	8 weeks per diet	TG reduced 3.1 % while on fish diet	TG and HDL-c increased by 7.9 % and HDL-c by 1.2 % while on red meat diet
	18–30 yrs old					
	*(TG – 60, Total-C - 173, LDL-C - 97)*	Vs.			Total-c and LDL-c reduced 2.3 % and 7.5 %, respectively, while HDL-c increased by 7.2 %, while on fish diet (sig)	
		Red meat diet (1.3 g n-3 PUFA)				
Baro (2003) [35]	15 males, 15 females (low background daily fish intake)	500 ml of n-3 PUFA enriched semi-skimmed milk (0.33 g EPA + DHA)	Intervention (no placebo, initial values vs. final)	4 week run in on low fish diet, 8 weeks consuming enriched milk	Total-c and LDL-c decreased 6 and 16 % (sig)	Homocysteine and VCAM-1 decreased by 13 % and 16 %, respectively
	Spanish					
	20–45 yrs old					
	*(TG – 108, Total-C - 176, LDL-C - 91)*					
Dyerberg (2006) and Dyerberg (2004) [27, 29]	79 males	Bakery products supplemented with 33 g of experimental fats: (a) 33 g control fat;	Randomized, double blind parallel arm trial	8 weeks	TG reduced 26 % from baseline in the n-3 PUFA group. Change was significantly greater than the TG reduction observed in the control group.	The n-3 PUFA diet resulted in a 3 beat/min reduction in heart rate of subject with a normal heart rate variability
	Danish					
	20–60 yrs old					
		Vs.			HDL-c reduced in the group receiving soy oil compared to the control	
	*(TG – 102, Total-C - 185, LDL-C - 116)*	(b) 12 g fish oil (4 g n-3 PUFA);				
		Vs.				
		(c) 33 g soy oil (20 g trans FA)				
Garcia-Alonso (2012) [36]	18 females	2 glasses of 250 ml n-3 PUFA-enriched tomato juice (500 mg EPA + DHA total)	Randomized, single blind, parallel arm trial	2 weeks	No effect on lipid profile	Enriched juice reduced serum homocysteine, VCAM-1 and ICAM-1 levels (sig)
	Spanish					
	35–55 yrs old	Vs.				
	*(TG – 59, Total-C - 197, LDL-C - 113)*	Placebo				
Hamazaki (2003) [37]	16 females, 25 males	1 glass of 250 ml Soybean milk enriched with:	Randomized, double blind placebo controlled trial	12 weeks	TG levels reduced 17 % (sig) in the group receiving the n-3 PUFA enriched soybean milk (no changes observed in the olive oil enriched milk)	
	Japanese					
	43–59 yrs old	Fish oil (0.6 g EPA + 0.26 g DHA)				
	*(TG – 154, Total-C - 211, LDL-C - 127)*	Vs.			LDL-c levels did not change, while total-c elevated in both groups by 2 %	
		Olive oil				
Coates (2009) [38]	29 males	200 g portion of pork from pigs fed a diet fortified with n-3 (0.185 g n-3 PUFA)	Randomized, double-blind, parallel arm, placebo controlled trial	12 weeks	TG levels reduced 27 % in the group consuming the n-3 PUFA fortified pork compared to controls	The n-3 PUFA fortified pork diet resulted in an elevation of serum thromboxane production (sig compared to the control)
	25–65 yrs old					
	*(TG – 84)*					
Stuglin (2005) [39]	15 males	3 flaxseed-enriched muffins (6.67 g ALA total)	Intervention (no placebo, compared initial and final values)	4 weeks	TG elevated 41 % (sig)	
	Canadian					
	22–47 yrs old					
	*(TG – 124, Total-C - 172, LDL-C - 108)*					
Dodin (2008) and Dodin (2005) [28, 30]	179 post-menopausal females	2 slices of flaxseed bread (8.42 g ALA)	Randomized, double blind, placebo controlled, parallel arm trial	12 months	Flaxseed-enriched bread raised the participants’ serum TG 3 %	Flaxseed bread reduced BMI from baseline values (sig)
	French Canadian					
	49–65 yrs old	Vs.			LDL-c reduced in the group receiving flaxseed bread compared to the placebo	
	*(TG – 101, Total-C - 221, LDL-C - 134)*					
		2 slices of ground grain bread				
Patenaude (2009) [40]	Group 1–10 females, 10 males	1 muffin, enriched with either:	Randomized, double blind, parallel arm trial	4 weeks	Diet (A) decreased total-c, LDL-c and TG by 7 %, 12 % and 11 % respectively, in Group 1. In group 2, Diet A decreased total-c and LDL-c 2 % while elevating TG by 13 %	Group 2 receiving diet B) had reduction in platelet aggregation (sig.)
	18–29 yrs old					
	*(TG – 91, Total-C - 165, LDL-C - 78)*	A) Ground flaxseed (6.5 g ALA)				
	Group 2–10 females, 10 males	Vs.				
	45–69 yrs old					
	*(TG – 81, Total-C - 181, LDL-C - 99)*	B) Flaxseed oil (5.74 g of ALA)			Diet (B) decreased TG 20 % in Group 1, while elevating TG by 3.5 % in Group 2	
Minihane (2005) [41]	19 males	n-3 PUFA-enriched cooking oil and margarine (2 g n-3 PUFA) with either:	Randomized, double blind, parallel arm trial	6 weeks	A diet containing a moderate ratio of n-6:n-3 PUFA resulted in 3 % and 8 % reductions in total-c and LDL-c, respectively, while increasing HDL-c by 8 % (0.05 < *p* < 0.1)	Diet providing a moderate ratio of n-6:n-3 PUFA increased total n-3 PUFA within RBC
	Indian Asian (in the UK)					
	35–70 yrs old					
						Diet providing a high ratio of n-6:n-3 PUFA increased plasma insulin levels and the participant’s HOMA-IR index (sig)
		Moderate n-6:n-3 (15 g n-6 PUFA)				
	*(TG – 140, Total-C - 192, LDL-C - 120)*					
		Vs.				
		High n-6:n-3 (26 g n-6 PUFA)				
Sofi (2013) [42]	12 males, 8 females	Gilthead sea bream fillets (2.3 g n-3 PUFA) fed either: Plant protein (2 g n-6 PUFA) Vs.	Randomized, single blind, cross-over trial	15 day run in with no fish consumption, 10 weeks on fishmeal fed fish followed by 10 weeks on plant protein fed fish (or vice versa)	TG, total-c and LDL-c decreased 11.7 %, 29.3 % and 21.6 %, respectively, in group first receiving fishmeal fed fish (sig). Values rebounded to normal following second dietary intervention	Group first receiving fishmeal fed fish experienced reductions in IL-6 and IL-8, and improvements in RBC filtrate rate
	Finish					
	23–67 yrs old					
	Group A: fish fed fishmeal followed by fish fed plant protein each for 10 weeks					
	*(TG – 117, Total-C - 233, LDL-C - 152)*					
		Fishmeal (1 g n-6 PUFA)				
					The group initially receiving plant protein fed fish experienced reductions in cholesterol occurring 10 weeks after subsequently fed fish fed fishmeal	
	Group B: fish fed plant protein followed by fish fed fishmeal each for 10 weeks					
	*(TG – 94, Total-C - 216, LDL-C - 139)*

**Table 2:** Alteration of serum TG levels in dietary intervention studies involving normolipidemic and moderately hyperlipidemic subjects

Study	N-3 PUFA Dose (g/d)	% Change in serum TG levels	*Duration (wks)*	*Additional Dietary Modifications*	Study	N-3 PUFA Dose (g/d)	% Change in serum TG levels	*Duration (wks)*	*Additional Dietary Modifications*
*Normolipidemic Subjects – Modified EPA and/or DHA Intake*					*Moderately Hyperlipidemic Subjects – Modified EPA and/or DHA Intake*				
Lara [31]	5.4	−15*	4	*Salmon Based*	Ambring [33]				
Dyerberg [27, 29]	4	−26*	8		Group A	4.1	−9*	4	*Mediterranean Based*
Hallund [32]					Group B	2.3	9	4	*Swedish Based*
Group A	3.4	−14	8	*Trout Based*	Sofi [42]				
Group B	0.8	−6	8	*Trout Based*	Group A	2.3	−2	10	*High LA diet*
Navas-Carretero [34]	2.8	−3.1	8	*Oily Fish Based*	Group B	2.3	−2	10	*Moderate LA diet*
Minihane [41]					Group C	2.3	−12	10	*Moderate LA diet*
Group A	2	3	6	*Moderate LA diet*	Group D	2.3	−2	10	*High LA diet*
Group B	2	−5	6	*High LA diet*	Hamazaki [37]	0.86	−17*	12	
Garcia-Alonso [36]	0.5	0	2						
Baro [35]									
Group A	0.33	2	8						
Group B	0.33	1	4						
Coates [38]	0.185	−27*	12						
*Normolipidemic Subjects – Modified ALA Intake*					*Moderately Hyperlipidemic Subjects – Modified ALA Intake*				
Stuglin [39]	6.98	41*	4	*Ground Flaxseed*	Dodin [28, 30]	8.53	3	52	*Ground Flaxseed*
Patenaude [40]									
Group A	6.5	−11	4	*Ground Flaxseed*					
Group B	6.5	13	4	*Ground Flaxseed*					
Group C	5.74	−20	4	*Flaxseed Oil*					
Group D	5.74	4	4	*Flaxseed Oil*					

*Asterisks denotes studies which found significantly different changes in serum TG levels (*p* < 0.05)

TG and LDL-c were only significantly lowered in interventions providing more than 4 g/day of n-3 PUFA through increased fish consumption. Two trials, of 4 weeks [31] and 8 weeks in duration [32], showed that fish consumption of 125–150 g/day (3.4-5.4 g/day of n-3 PUFA) reduced TG levels by 14-15 %. The 4-week intervention also showed a statistically non-significant 7 % reduction in LDL-c [31]. Another study demonstrated that switching from a Swedish diet to a Mediterranean diet (2.3 g vs. 4.1 g/day of n-3 PUFA) for 4 weeks resulted in statistically significant 17 %, 23 % and 17 % reductions in serum total-c, LDL-c and TG, respectively [33]. Finally, participants consuming an oily fish diet (2.6-3.0 g/day of n-3 PUFA) for 8 weeks experienced non-significant reductions in total-c and LDL-c by 2.3 % and 7.5 %, respectively, and a trend towards lowered TG levels (by 3.1 %) was observed when compared to a red meat diet (1.2-1.4 g/day of n-3 PUFA) [34].

There were five studies designed to increase EPA and/or DHA consumption through enriched baked goods [27, 29], drinks [35–37], or pork derived from animals consuming marine based n-3 PUFA-enriched food [38]. Two 12 week studies, one providing 0.86 g/day of n-3 PUFA from enriched milk and the other providing 0.185 g/day of n-3 PUFA from enriched pork, produced significant reductions in serum TG of 17 % and 27 %, respectively, while not significantly altering serum cholesterol levels [37, 38]. One 8-week intervention providing 0.33 g/day of n-3 PUFA from enriched milk showed a reduction in total-c and LDL-c of 6 % and 16 %, respectively [35]; while a second 8-week study providing 4 g/day of n-3 PUFA from enriched baked goods produced a statistically significant 26 % reduction in TG levels [27, 29]. In contrast, a 2-week study providing 0.5 g/day of n-3 PUFA from EPA/DHA enriched tomato juice did not report any changes in TG or cholesterol levels, however, participants show significant reductions in circulating homocysteine, VCAM-1 and ICAM-1 [36].

Studies assessing an increased consumption of ALA utilized 30–40 g/day of flaxseed (5.74-8.42 g/day of ALA) [28, 30, 39, 40], yet the results from these trials produced inconsistent effects on lipid levels. For instance, a 4-week intervention with 32.7 g/day of ground flaxseed led to a 41 % increase in TG levels [39]. In contrast, a 12-month intervention, providing 40 g/day of flaxseed, resulted in the maintenance of cholesterol and TG levels, while the placebo significantly elevated plasma total-c and LDL-c levels [28, 30]. Additionally, one 4-week intervention that provided participants with 5.74-6.5 g/day of ALA found non-significant reductions in serum lipids [40]. Interestingly, although non-significant, the consumption of ground flaxseed or flaxseed oil (each providing an equal amount of ALA) resulted in 11 % and 20 % reductions in TG levels, respectively, in individuals 18–29 years of age; ground flaxseed also led to 7 % and 12 % reductions in total-c and LDL-c, respectively [40].

All of the previously mentioned studies reduced an individual’s dietary n-6:n-3 PUFA ratio by elevating n-3 PUFA consumption. However, two studies investigated the effects of altering the n-6:n-3 PUFA dietary ratio by increasing n-6 PUFA intake while maintaining a constant n-3 PUFA intake. This allowed for the investigation into whether a reduced n-6:n-3 PUFA ratio alone is more beneficial than an absolute increase in n-3 PUFA consumption as well as a decreased dietary n-6:n-3 PUFA ratio, as evaluated in the previous studies. One study accomplished this by replacing monounsaturated fatty acids with n-6 PUFA; participants either consumed a moderate or high ratio of n-6:n-3 PUFA consisting of 15 g or 26 g/day, respectively, of n-6 PUFA while consistently consuming 2 g/day of n-3 PUFA for 6 weeks [41]. While there were no statistically significant differences between treatments, the diet providing 15 g of n-6 PUFA produced a trend towards reduced total-c and LDL-c by 3 % and 8 %, respectively, and a trend towards elevated HDL-c by 8 % [41]. During a 10-week cross-over study, participants consumed 90 g/day of fishmeal- or plant protein-fed gilthead sea bream that provided either 1 g or 2 g/day of n-6 PUFA, respectively, while consistently providing 2.3 g/day of n-3 PUFA [42]. This study showed that individuals who first consumed the fishmeal-fed gilthead sea bream had a reduction in total-c, LDL-c and TG by 29.3 %, 21.6 % and 11.7 %, respectively, prior to rebounding after 10 weeks of consuming plant protein-fed fish [42]. However, participants who first consumed the plant protein-fed fish did not experience reductions in any lipid markers following either dietary intervention, except for a 5 % reduction in total-c following the cross over period [42].

### Supplementation studies

Using the selection criteria previously described, 22 clinical trials (1637 subjects in total) utilizing n-3 PUFA in a supplement form were evaluated in the current review (Table 3). These trials assessed the effect of n-3 PUFA supplementation on blood lipid profiles in individuals with normal and borderline high levels of TG, total-c, and LDL-c. These studies included participants from ages 18 to 75 years, with a treatment duration ranging from 2 to 52 weeks. The main source of n-3 PUFA from these studies was fish oil [43–59], which provided an approximate EPA:DHA ratio of 1.5:1. Five studies utilized a DHA-rich oil from an algal source [59–63], and two studies employed an EPA-rich oil from fish sources [59, 64]. The studies encouraged participants to consume n-3 PUFA supplements in the form of 1 to 12 capsules per day while maintaining normal dietary habits. Supplementation provided 0.3-4.9 g/day of n-3 PUFA. As shown in Table 4, fasting TG levels were reduced following supplementation with EPA and/or DHA exclusively in 30/34 experimental arms within the 22 studies (i.e. 88 % of the interventions evaluated, without taking statistical significance into account) and post-supplementation TG levels were significantly lowered from baseline in 23 of the 34 aforementioned experimental arms (i.e. 68 % of the interventions; however, one study did not analyze for an in-group difference in serum lipid levels from baseline to post-supplementation [62]).

**Table 3:** Studies assessing the lipid lowering effects of n-3 PUFA utilizing a supplement

Study	Subject Characteristics	n-3 PUFA Source and Dose	Study Design	Duration	Lipid Outcomes	Other Findings
	*Average baseline TG, Total-C, LDL-C (mg/dL)*					
Fakhrzadeh (2010) [43]	73 females, 51 males	1 capsule of fish oil	Randomized, double-blind, placebo controlled, parallel arm	26 weeks	TG levels increased 15 % in the placebo group and decreased 2 % in the treatment group (sig. between group effect)	
	Age: 65+					
	*(TG – 145, Total-C - 190, LDL-C - 114)*	0.18 g EPA + 0.12 g DHA			LDL-c, HDL-c, or total-c did not change	
		Vs.				
		Placebo				
Sanders (2011) [44]	225 female, 142 males	3 capsules of fish oil (1:5 ratio of EPA:DHA) containing:	Randomized, placebo controlled, parallel arm, double-blind	52 weeks	TG levels reduced 16.5 % by 1.8 g/day, and was unchanged in both 0.45 g/day and 0.9 g/day (sig)	No change in blood pressure, arterial stiffness, or measures of endothelial function after supplementation
	Age: 45–70					
	*(TG – 100, Total-C - 210, LDL-C - 125)*	a) 0.45 g n-3 PUFA				
		Vs.				
		b) 0.9 g n-3 PUFA			Total-c , HDL-c and LDL-c was unchanged after supplementation at each dose	
		Vs.				
		c) 1.8 g n-3 PUFA				
		Vs.				
		Placebo				
Hlais (2013) [45]	112 males	Fish oil (FO) capsules (Per gram: 0.737 g of n-3 PUFA: 0.495 g EPA + 0.196 g DHA):	Randomized, single blind, parallel arm study	6 and 12 weeks	*After 6 weeks :*	No significant effects on glycemic and blood pressure parameters were noted
	Age: 18–35					
	(*TG – 125, Total-C - 187, LDL-C - 118)*	A) 2 g of FO			TG was reduced by 15 %, 4 %, 10 % in Groups A, B, D, respectively, and elevated by 6 %, 3 % in Groups C, E, respectively (only group A was sig)	
		Vs.				
		B) 1 g of FO and 8 g of sunflower oil				
		Vs.			Total-c and LDL-c was elevated by 2-8 % in Groups A, B, C, E (non-sig) and by 7 % and 13 % in Group D, respectively (sig)	
		C) 2 g of FO and 8 g of sunflower oil				
		Vs.			HDL-c was elevated by 4-6 % in Groups A, C, D and reduced by 10 % and 3 % in Groups B, E, respectively (non-sig)	
		D) 4 g of FO and 8 g of sunflower oil				
		Vs.				
					*After 12 weeks :*	
					TG was reduced by 12 %, 12 %, 2 %, 5 % in Groups A, B, D, E respectively, and elevated by 1 % in Groups C (non-sig)	
					Total-c was elevated by 4 % in Groups A, D, and reduced by 1 %, 4 % and 10 % in groups B, C, E, respectively (only Group E was sig)	
		E) 8 g of sunflower oil			LDL-c was elevated by 3-7 % in Groups A, B, D (non-sig) and reduced by 5 % and 13 % in Group C, E, respectively (only Group E was sig)	
					HDL-c was elevated by 6 %, 2 % in Groups A, D, respectively, and reduced by 5-7 % in Groups B, C, E, (non-sig)	
Nilsson (2012) [46]	28 females, 10 males	5 capsules of fish oil	Randomized, placebo controlled, crossover study	5 weeks	TG reduced 12 % (sig)	Systolic and Diastolic blood pressure was reduced by 5 %
	Age: 51–72	1.5 g EPA, 1.05 g DHA, and 0.45 g of other n-3 PUFA				
	*(TG – 142)*					
						Inflammatory markers were unchanged
		Vs.				
		Placebo				
Rizza (2009) [47]	25 females, 25 males	2 capsules of fish oil (0.6 g EPA, 0.4 g DHA per capsule)	Randomized, double-blind, parallel designed, placebo controlled	12 weeks	TG levels reduced 26 % with treatment (sig)	Improvement in flow mediated dilation in treatment group
	Age: 29.9+/− 6.6					
	*(TG – 118, Total-C - 192, LDL-C - 122)*	Vs.			HDL-c, LDL-c and total-c did not change	
		Placebo				
Lovegrove (2004) [48]	84 Males	4 capsules of fish oil	Randomized, double bind, placebo controlled, parallel arm	12 weeks	TG was reduced 31 % (sig)	
	Age: 30–70	1.5 g EPA, 1.0 g DHA			HDL-c increased (sig)	
	(*TG – 128, Total-C - 207, LDL-C - 128)*	Vs.			No effect on total-c or LDL-c was observed	
		Placebo				
Ciubotaru (2003) [49]	30 Post Menopausal Females on Hormone Replacement Therapy	Fish oil	Randomized, double blind, placebo controlled	5 weeks	TG reduced 26 % in group receiving 14 g of fish oil (sig) and 4 % in group receiving 7 g of fish oil	CRP reduced, IL-6 reduced in groups receiving fish oil supplements
		Randomized to three groups:				
	Age: 60+/− 5	a) 14 g safflower oil (0 g EPA/DHA)				
	(*TG – 121, Total-C - 220, LDL-C - 126)*					
		Vs.			Group receiving safflower oil alone experienced a 21 % increase in TG levels	
		b) 7 g safflower oil + 7 g fish oil (1.45 g EPA + DHA)				
					No change in LDL-c or total-c	
		Vs.				
		14 g fish oil				
		(2.9 g EPA+ DHA)				
Offman (2013) [50]	15 Females, 37 Males	Fish oil: 4 g of Epanova or 4 g of Lovaza	Open label, parallel group cohorts	2 weeks	Participants receiving Epanova had reductions in TG, HDL-c and LDL-c of 21 %, 5 % and 4 %, respectively	Epanova raised plasma total EPA + DHA concentrations 3 times the level as subject’s receiving Lovaza
	Age: 18–55					
	(*TG – 166, Total-C - 189, LDL-C - 128)*	Lovaza: 1.8 g of EPA, 1.5 g of DHA				
					Participants receiving Lovaza had reductions in TG and HDL-c levels of 8 % and 7 %, respectively, while raising LDL-c by 0.4 %	
		Epanova: 2.2 g of EPA and 0.8 g of DHA				
					(effects on TG between groups were significant)	
Laidlaw (2003) [51]	31 Females	Fish oil capsules (4 g of EPA + DHA) with:	Randomized, parallel arm study	4 weeks	TG was reduced 35-40 % in groups receiving 0 g, 1 g and 2 g of GLA (sig), and TG was reduced 7 % in the group receiving 4 g of GLA	
	Age: 36–68					
	(*TG – 112, Total-C - 213, LDL-C - 134)*	0 g of gamma-linolenic acid (GLA)				
		Vs.			All groups had reductions in total-c of 1-9 %	
		1 g of GLA			LDL-c was reduced in all groups by 2-13 %, except in the group receiving 1 g of GLA (only sig in the group receiving 2 g of GLA)	
		Vs.				
		2 g of GLA				
		Vs.				
		4 g of GLA				
Mann (2010) [52]	19 Females, 11 Males	10 capsules containing:	Randomized, double-blind, parallel designed study	2 weeks	TG was reduced 25 % in the group receiving Seal oil and 21 % in the group receiving Tuna oil (sig)	CRP was reduced by 11 % and 25 % in the groups receiving tuna oil and fish oil, respectively
	Age: 20–50	Tuna oil (0.21 g of EPA, 0.03 g of DPA, 0.81 g of DHA)				
	(*TG – 120, Total-C - 196, LDL-C - 134)*					
		Vs.				
		Seal oil (0.34 g of EPA, 0.23 g of DPA, 0.45 g of DHA)			LDL-c was elevated through both interventions by 3 %	
		Vs.				
		Placebo				
Vanschoonbeek (2004) [53]	20 Males	9 capsules of fish oil:1.05 g EPA, 0.75 g, DHA, and 1.2 g other n-3 PUFA	Intervention (no placebo, compared initial vs. final values)	4 weeks	TG was reduced 10 % (sig)	Treatment lowered integrin activation, as well as plasma levels of fibrinogen and factor V
	Age: 48.5+/− 9.8				Total-c was unchanged	
	*(TG – 141, Total-C - 218, LDL-C - 151)*				LDL-c increased 5 % and remained borderline high	
Di Stasi (2004) [54]	18 Females, 18 Males	Fish Oil Capsules (46 % and 39 % of n-3 PUFA was EPA and DHA, respectively):	Randomized, parallel arm study	12 weeks	There was no significant change in TG levels from baseline within each group, however, a significant dose response was noted. n-3 PUFA provided at 2 g and 4 g per day resulted in TG reductions by 15 and 20 %, respectively.	
	Age: 21–51					
	(*TG – 87, Total-C - 211)*					
		1 g of n-3 PUFA/day				
		Vs.				
		2 g of n-3 PUFA/day				
		Vs.				
		4 g of n-3 PUFA/day				
Stark (2000) [55]	35 Postmenopausal Females	8 Fish Oil capsules:	Randomized, double blind, placebo controlled, cross-over study	4 weeks	n-3 PUFA produced a 26 % reduction in serum TG levels (sig)	
		2.4 g EPA + 1.6 g of DHA			n-3 PUFA produced a 5 % increase in LDL-c levels	
	Age: 43–60	Vs.				
	(*TG – 120, Total-C - 213, LDL-C - 122)*					
		Placebo (primrose oil)				
Damsgaard (2008) [56]	66 males	10 capsules of fish oil (2.0 g EPA, 1.25 g DHA)	Randomized, double-blind placebo controlled, 2x2 factorial design	8 weeks	TG levels were reduced 19 % with high LA intake (sig) and 51 % reduction with low LA intake (sig)	-No change in inflammatory markers
	Age: 19–40					
	*(TG – 89, Total-C - 153, LDL-C - 99)*	Vs.				
		Placebo				
		Supplementation with either high LA in diet or low LA in diet			No changes in HDL-c, LDL-c, total-c	
Brady (2004) [57]	29 Males	fish oil capsules	Double-blind, parallel, dietary intervention	6 weeks	TG was reduced 20 % and 25 % in high and moderate groups, respectively (sig)	
	Age: 35–70	(2.5 g EPA+ DHA) with either:				
		Moderate n-6 PUFA diet (olive oil)				
					No changes in HDL-c, LDL-c, total-c	
	*(TG – 137, Total-C - 186, LDL-C - 114)*					
		Vs.				
		High n-6 PUFA diet (corn oil)				
Kaul (2008) [58]	54 females, 34 males	2, 1 g capsules per day containing	Randomized, double blind, placebo controlled, parallel arm study	12 weeks	Fish oil produced a 4 % and 7 % increase in total-c and LDL-c, respectively	No significant change in CRP or TNF-ɑ levels
	Age: 31–36					
	(*TG – 113, Total-C - 184, LDL-C - 102)*	Fish oil (0.606 g of n-3 PUFA; 0.242 g of DHA + 0.352 g of EPA)			Flaxseed oil produced a 4 % and 12 % increase in total-c and LDL-c, respectively	No significant change in platelet aggregation stimulated by thrombin or collagen
					Hempseed oil produced a 4 % increase in both total-c and LDL-c and an 18 % increase in TG	
		Vs.				
		Flaxseed oil (1.02 g of ALA)				
		Vs.				
		Hempseed oil (0.372 g of ALA,1.14 g of LA)				
		Vs.				
		Sunflower oil (1.36 g of LA)				
Buckley (2004) [59]	20 Females, 22 Males	9 capsules of EPA or DHA-rich oil	Randomized, double bind, placebo controlled, parallel arm	4 weeks	EPA treatment: TG decreased 22 % (sig)	
	Age: 20–70					
		4.8 g EPA				
	*(TG – 106, Total-C - 205, LDL-C - 124)*	Vs.			DHA treatment: TG decreased 38 % (sig)	
		4.9 g DHA			Total-c decreased (*p* = 0.06)	
		Vs.				
		Placebo			No changes in LDL-c or HDL-c	
Sanders (2006) [60]	40 Females, 39 Males	4 capusles of DHA-rich oil from *Schizochytrium sp.*	Randomized double bind, placebo controlled, parallel arm	4 weeks	TG decreased from baseline 14 % (sig)	
	Age: 31 +/− 14				No changes in total-c or LDL-c levels	
	(*TG – 89, Total-C – 175, LDL-C – 96)*	1.5 g DHA + 0.6 g DPA				
		Vs.			HDL-c increased by 9 % (sig)	
		Placebo				
Stark (2004) [61]	32 Postmenopausal Females	12, 500 mg Capsules containing DHA from an algal source	Randomized, double blind, placebo controlled, cross-over study	4 weeks	DHA decreased TG by 8 % (sig).	DHA was able to reduce resting heart rate by 7 %
	Age: 45–70					
		2.8 g of DHA			DHA elevated HDL-c by 8 %, total-c by 4 % and reducing LDL-c by 8 %	
	(*TG - 132, Total-C – 216, LDL-C – 125)*	Vs.				
		Placebo (corn and soy oil mixture)				
Wu (2006) [62]	25 Postmenopausal Vegetarian Females	Capsules providing 6 g of DHA rich algae oil	Randomized, single blind, placebo controlled study	6 weeks	DHA decreased TG by 18 %, Total-c by 3 % and LDL-C by 3 % while elevating HDL-C by 6 %	No changes in levels of urinary estrogen metabolites, or markers of oxidative stress (e.g. ɑ-tocopherol)
	Age: 52 +/− 5 yrs	2.4 g of DHA				
	*(TG - 124, Total-C – 158, LDL-C - 90)*					
		Vs.			*Only between group analysis was performed	
		Placebo (corn oil)				
Geppert (2006) [63]	87 Females, 87 Males Vegetarians	4 capusles of DHA-rich oil from *Ulkenia sp.*	Randomized, double blind, parallel design, placebo controlled	8 weeks	TG reduced 23 % in the group receiving DHA rich oil (sig).	No significant changes in haemostatic factors
	Age: 28–43	0.94 g of DHA			Total cholesterol, LDL-c and HDL-c increased 6-11 % in the group consuming DHA rich oil (sig)	
		Vs.				
	(*TG - 95, Total-C – 180, LDL-C – 97)*					
		Placebo (olive oil)				
					No changes in the placebo group	
Cazzola (2007) [64]	93 Young Males, 63 Older Males	9 capsules of EPA-rich fish oil containing either:	Randomized, double blind, placebo controlled	12 weeks	TG levels were reduced ~25 % after 1.35, 2.7 or 4.05 g of EPA across all ages (sig)	EPA supplementation tended to decrease soluble ICAM-1
		a) 1.35 g EPA				
	Age: Young, 18–42; Old, 53–70	Vs.				
		b) 2.7 g EPA				
	(*TG - 82, Total-C – 162, LDL-C – 103)*	Vs.			No effect on HDL-c, LDL-c, or total-c in any group	
		c) 4.05 g EPA				
		Vs.				
		Placebo

**Table 4:** Alteration of serum TG levels in supplementation studies involving normolipidemic and moderately hyperlipidemic subjects

Study	N-3 PUFA Dose (g/d)	% Change in serum TG levels	*Duration (wks)*	*Additional Supplement (g/d)*	Study	N-3 PUFA Dose (g/d)	% Change in serum TG levels	*Duration (wks)*	*Additional Supplement (g/d)*
*Normolipidemic Subjects - EPA and/or DHA Supplements*					*Moderately Hyperlipidemic Subjects – EPA and/or DHA Supplements*				
Cazzola [64]					Buckley [59]				
Group A	4.05	−30*	12		Group A	4.9	−8*	4	
Group B	4.05	−25*	12		Group B	4.8	−22*	4	
Group C	2.7	−25*	12		Laidlaw [51]				
Group D	2.7	−33*	12		Group A	4	−40*	4	
Group E	1.35	−22*	12		Group B	4	−39*	4	*1 g of* γ*-linolenic acid*
Group F	1.35	−33*	12		Group C	4	−35*	4	*2 g of* γ*-linolenic acid*
Damsgaard [56]					Group D	4	−7	4	*4 g of* γ*-linolenic acid*
Group A	3.25	−51*	8	*Low LA diet*	Di Stasi [54]				
Group B	3.25	−19*	8	*High LA diet/Olive Oil*	Group A	4	−20	12	
Nilsson [46]	3	−12*	5		Group B	2	−15	12	
Hlais [45]					Group C	1	9	12	
Group A	2.95	−10	6	*8 g of Sunflower Oil*	Stark [55]	4	-26*	4	
Group B	2.95	−2	12	*8 g of Sunflower Oil*	Offman [50]				
Group C	1.47	−15*	6		Group A	3.3	−8	2	
Group D	1.47	6	6	*8 g of Sunflower Oil*	Group B	3	−21	2	
Group E	1.47	−12	12		Vanschoonbeek [53]	3	−10*	4	
Group F	1.47	1	12	*8 g of Sunflower Oil*	Ciubotaru [49]				
Group G	0.74	−4	6	*8 g of Sunflower Oil*	Group A	2.9	−26*	5	
Group H	0.74	−12	12	*8 g of Sunflower Oil*	Group B	1.45	−4	5	*7 g of Safflower Oil*
Brady [57]					Stark [61]	2.8	−20*	4	
Group A	2.5	−25*	6	*Moderate LA diet/Olive Oil*	Lovegrove [48]	2.5	−31*	12	
Group B	2.5	−20*	6	*High LA diet - Corn Oil*	Sanders [44]				
Wu [62]	2.4	−18*	6		Group A	1.8	−17*	52	
Sanders [60]	2.1	−21	4		Group B	0.9	0	52	
Rizza [47]	2	−26*	12		Group C	0.45	0	52	
Geppert [63]	0.94	−23*	8		Mann [52]				
Kaul [58]					Group A	1.05	−21*	2	
Group A	0.61	0	12		Group B	1.02	−25*	2	
					Fakhrzadeh [43]	0.3	−2	26	
*Normolipidemic Subjects - ALA Supplements*									
Kaul [58]									
Group B	1.02	0	12	*Flaxseed Oil; 0.28 g of LA*					
Group C	0.37	15	12	*Hempseed Oil; 1.02 g of LA*					

*Asterisks denotes studies which found significantly different changes in serum TG levels (*p* < 0.05)

The magnitude of the n-3 PUFA-mediated TG-lowering effect varied depending on the supplementation dose and the study duration. Low doses such as 0.3-0.9 g/day of n-3 PUFA for 12–52 weeks did not consistently produce a significant reduction in TG levels [43–45, 63]. While studies solely providing n-3 PUFA in doses < 1 g/day (EPA, DHA or both) significantly lowered fasting TG by 8-38 % [44–55, 59, 61, 62, 64]. In a dose–response study, 1.8 g/day of EPA and DHA for 52 weeks was sufficient to lower TG levels by 16.5 %, whereas 0.45 and 0.9 g/day did not affect fasting TG levels [44]. A second study demonstrated a significant dose–response effect whereby 1 g/day of marine derived n-3 PUFA produced an elevation in TG levels by 9 %, while 2 and 4 g/day of n-3 PUFA for 12 weeks produced decreases in TG levels of 15 % and 20 %, respectively [54]. However, none of the aforementioned doses produced a significant change from baseline values [54].

Two studies investigated the effect of fish oil based n-3 PUFA supplementation combined with a background diet that was either high or moderate in n-6 PUFA, specifically linoleic acid (18:2 n-6, LA) [56, 57]. In an 8 week study, 3.1 g/day of n-3 PUFA resulted in a 19 % and a 51 % reduction in TG levels while consuming either a high or moderate n-6 PUFA background diet, respectively [56]. Similarly, participants of a 6 week study, stratified to a high or moderate LA background diet found that 2.5 g/day of EPA and DHA benefited both groups with similar reductions in TG levels of 20 % and 25 %, respectively [57].

Studies that utilized an algal source of DHA consistently demonstrated the TG-lowering effects of the supplement [59–63]. Three 4-week studies, providing 1.5, 2.8 and 4.9 g/day of DHA, found significant reductions in TG levels of 14 %, 8 % and 38 %, respectively [59–61]. Further, a 6-week study providing 2.4 g/day of algal-derived DHA showed a significant 18 % reduction in TG levels compared to a placebo [62]. Additionally, a study providing only 0.94 g/day of DHA, but for 8 weeks, noted a significant 23 % reduction in TG levels [63].

Blood cholesterol levels were also examined in the 22 supplementation studies within the current review, and only two studies [48, 60] observed modest n-3 PUFA-induced increases in HDL-c levels that were significantly different from baseline. Thus, total-c, LDL-c, and HDL-c remained largely unchanged with n-3 PUFA supplementation. A modest reduction in total-c occurred with 4.9 g/day of DHA, yet this difference was not statistically significant [59]. Additionally, only 1 study produced a significant 11 % reduction in LDL-c levels following 4 weeks of supplementation with 4 g/day of n-3 PUFA, however, this effect was produced with an additional 2 g/day supplement of gamma-linolenic acid (18:3 n-6, GLA) [51].

## Discussion

This review indicates that the established TG-lowering effect of n-3 PUFA in hyperlipidemic individuals is maintained within populations who are normolipidemic to borderline hyperlipidemic. Studies in which participants consumed EPA and/or DHA or fish consistently reported lower blood TG levels in comparison to those studies in which participants consumed plant-based sources of n-3 PUFA. Overall, the studies involving dietary interventions evaluated within the current review suggest that a TG-lowering effect of marine based n-3 PUFA is produced in healthy individuals upon the consumption of ≥ 4 g/day of n-3 PUFA. In contrast, the supplementation studies assessed within the current review suggest that a minimum of 1 g/day of EPA and/or DHA (derived from either fish or algal oil) is required to confer a similar benefit as observed in the dietary interventions.

Within the past 5 years, the European Food Safety Authority (EFSA), the AHA and the Food Standards - Australia and New Zealand (FSANZ) organization have all recognized n-3 PUFA as a preventative measure against the development of CVD, primarily by reducing risk factors for CVD, including elevated blood TG levels [12–15]. The EFSA established and substantiated a health claim in 2010 indicating the consumption of 2 g of EPA/DHA per day has the ability to maintain normal blood TG concentrations [12, 13]. Furthermore, the AHA released a statement in 2011 indicating that a daily dosage of 2–4 g of n-3 PUFA, specifically EPA and DHA, confers a 25-30 % decrease in serum TG levels [14].

The health claim by the EFSA and the statement by the AHA are largely based on the findings of a systematic review by W.S. Harris [19]. This review stratified studies comparing participants with either healthy (serum levels < 177 mg/dL) or elevated levels (serum levels ≥ 177 mg/dL) of serum TG; relative to the upper limit for serum TG of 200 mg/dL utilized in the current review [19]. However, in the review by Harris, a quarter of the studies assessing participants with healthy baseline TG levels contained a hypercholesterolemic population (serum TC ≥ 240 mg/dL). Nonetheless, the results indicated that 3–4 g/day of EPA and/or DHA produced a 25-34 % decrease in serum TG levels [19]. This finding is supported by a later systematic review which reported that individuals with either borderline high or high levels of serum TG (according to AHA guidelines) experienced reductions in TG levels of ~20 % and ~30 %, respectively, when consuming 4 g/day of EPA and/or DHA [18].

The previous reviews indicate that marine based n-3 PUFA can reduce serum TG levels; however, they primarily focused on supplementation studies within dyslipidemic populations. An earlier review by W. S. Harris, than the 1997 study previously discussed, included dietary intervention studies and produced findings similar to those of the current review. Overall, consuming n-3 PUFA through dietary forms primarily showed a trend in TG reductions, while a significant effect was only observed when large amounts of n-3 PUFA were consumed (as observed in Table 2, ≥ 4 g/day of n-3 PUFA) and the effects of ALA supplementation were highly variable [17]. W. S. Harris concluded that this inconsistency in the ability of n-3 PUFA to reduce serum TG levels during dietary interventions was likely due to the manipulation of multiple variables as the food source was not highly controlled between studies [19]. The lack of a consistent study design for elevating n-3 PUFA consumption through dietary modifications continues to be a limitation for the field. This constraint reduces the ability to evaluate an exact dosage and source of EPA and/or DHA required to significantly, and routinely, lower serum TG levels across all populations.

**Fig. 2:**
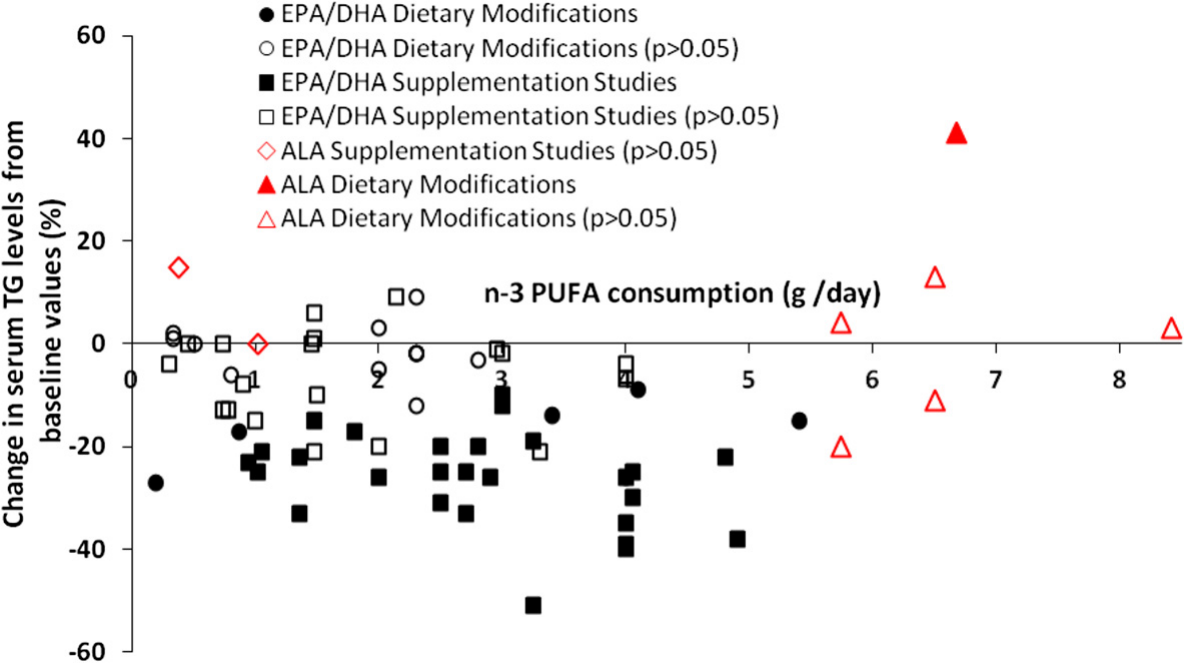
The percent change in serum TG levels from baseline values in normolipidemic and borderline hyperlipidemic subjects receiving n-3 PUFA either through the diet or supplemental forms. Shaded markers indicate changes from baseline that are statistically significant (*p* < 0.05)

Several reviews have repeatedly shown an effect of EPA and/or DHA in lowering TG levels during supplementation trials [17, 19–24]. Based on the current review, when ≥ 1 g/day of EPA and/or DHA is consumed by individuals, without any other increases in dietary fat intake, an 8-40 % reduction in TG levels can be observed, as shown in Table 4. The apparent presence of a lipid-lowering dose–response to marine derived n-3 PUFA intake, demonstrated in the study by Di Stasi *et al.* [54], suggests additional benefits are attainable when supplementation is raised as high as 4.9 g/day [54]. Figure 2 summarizes the TG-lowering effects of individuals consuming ALA, EPA, DHA or some combination of these n-3 PUFA within the studies analyzed in this review. This figure highlights the consistent lipid-lowering effect of EPA and DHA; studies providing ALA remain inconclusive. Additionally, Fig. 2 indicates that as the dose of EPA and/or DHA increases, in both supplementation and dietary intervention studies, a concurrently larger reduction in TG levels is obtained. Furthermore, Fig. 2 shows neither EPA and/or DHA significantly raised TG levels from baseline in either supplementation or dietary intervention studies.

Based on the present review, the beneficial effects of n-3 PUFA, specifically EPA and DHA, which have been substantiated in hyperlipidemic individuals, extend to individuals with normal to borderline high levels of serum lipids. In summary, using select search terms and criteria, our review of the existing evidence has shown that consumption of ≥ 4 g/day of n-3 PUFA through marine and EPA and/or DHA-enriched food sources, or 1–5 g/day of EPA and/or DHA in supplement form, has the ability to reduce serum TG by 9-26 % and 4-51 %, respectively, in normolipidemic to borderline hyperlipidemic and otherwise healthy individuals. This provides evidence that the consumption of marine based n-3 PUFA is not only extremely useful to treat dyslipidemia, but is also beneficial for otherwise healthy populations in the prevention of hyperlipidaemia and may subsequently reduce the risk of developing CVD.

## Abbreviations

PUFA: Polyunsaturated fatty acids
TG: Triacylglycerol
LDL-c: Low density lipoprotein cholesterol
HDL-c: High density lipoprotein cholesterol
Total-c: Total cholesterol
FO: Fish oil
ALA: Alpha-linolenic acid
EPA: Eicosapentaenoic acid
DPA: Docosapentaenoic acid
DHA: Docosahexaenoic acid
LA: Linoleic acid
AA: Arachidonic acid
GLA: Gamma-linolenic acid
AHA: American Heart Association
EFSA: European Food Safety Authority
FSANZ: Food Safety – Australia and New Zealand
